# Cerebrotendinous xanthomatosis in Slovak patients – experience with clinical manifestations and diagnostic approaches

**DOI:** 10.1007/s10048-026-00902-6

**Published:** 2026-04-18

**Authors:** Pavol Ďurina, Andrej Bandura, Ján Chandoga, Miriama Juhosová, Marcel Repiský, Petra Jungová, Jana Roháľová, Dominika Jarásková, Viktória Pohorelská, Silvia Dallemule, Slavomíra Mattošová, Katarína Brennerová, Katarína Okaľová, Imre Bohuniczky, Daniel Böhmer

**Affiliations:** 1 Department of Molecular and Biochemical Genetics, Institute of Medical Biology, Genetics and Clinical Genetics, Faculty of Medicine of Comenius University, University Hospital Bratislava, Mickiewiczova 13, Bratislava, 813 69 Slovakia; 2 Faculty of Medicine, Institute of Medical Biology, Genetics and Clinical Genetics, Comenius University, Sasinkova 4, Bratislava, 811 08 Slovakia; 3 Faculty of Natural Sciences, Department of Physical and Theoretical Chemistry, Comenius University, Ilkovičova 3220/6, Bratislava, 842 15 Slovakia; 4 Department of Neurology, Central Military Hospital, gen. Miloša Vesela 21, Ružomberok, 03426 Slovakia; 5 Department of Paediatrics, Faculty of Medicine of Comenius University and National Institute for Children’s Diseases, Limbová 1, Bratislava, SK-83340 Slovakia; 6 Pediatric Clinic, Banska Bystrica Children’s University Hospital, Banská Bystrica, 974 09 Slovakia; 7 The Department of Paediatric Ophthalmology, Comenius University and National Institute of Children’s Diseases, Limbová 1, Bratislava, 833 40 Slovakia

**Keywords:** Cerebrotendinous xanthomatosis, Cataract, Central Europe, Cholestanol, 7-dehydrocholesterol

## Abstract

Cerebrotendinous xanthomatosis (CTX) is a rare autosomal recessive disorder of bile acid synthesis caused by pathogenic variants in *CYP27A1*. The disease is characterized by progressive neurological impairment, cataracts, and tendon xanthomas and is biochemically defined by the accumulation of cholestanol, cholesterol precursors, and bile alcohols. We report the first Slovak cohort of eight CTX patients diagnosed using combined biochemical and molecular genetic approaches. Four adult patients were referred for laboratory investigation due to neurological manifestations, predominantly ataxia and spastic paraparesis, with diagnosis established in the fourth decade of life. Among the remaining individuals, two children and one adolescent were diagnosed based on cataracts or neurological symptoms, while one middle-aged woman was identified through family screening and presented mainly with psychiatric manifestations. All patients exhibited markedly elevated serum cholestanol concentrations, consistently accompanied by increased 7-dehydrocholesterol as a side finding. Molecular analysis identified six pathogenic variants (c.379 C > T, c.819delT, c.1016 C > T, c.1183 C > T, c.1184 + 1G > A, and c.1263 + 5G > A), including the intronic variant c.1263 + 5G > A, which has not been previously reported in CTX patients. The clinical manifestations observed in this cohort illustrate the broad phenotypic variability of CTX and reflect diagnostic process leading to disease recognition. Increased awareness of these clinical manifestations may prompt earlier laboratory evaluation using biochemical and molecular genetic testing.

**Supplementary Information:**

The online version contains supplementary material available at 10.1007/s10048-026-00902-6.

## Introduction

Cerebrotendinous xanthomatosis (CTX) is a rare autosomal recessive metabolic disease, presenting with a large spectrum of neurological symptoms, cataracts, and tendon xanthomas. Van Bogaert and colleagues described CTX for the first time in 1937, in two patients with cognitive and motor impairment combined with cataracts and xanthomas [1]. Since then, over 500 patients worldwide have been reported in the literature. CTX is caused by biallelic pathogenic variants in the *CYP27A1* gene, which encodes mitochondrial sterol 27-hydroxylase, an enzyme involved in bile acid synthesis [2, 3]. Enzyme deficiency results in reduced bile acid production and compensatory up-regulation of the 7α-hydroxylation and 25-hydroxylation pathways, leading to accumulation of sterol intermediates and metabolites in biological fluids, including bile alcohols, 7α-hydroxycholest-4-en-3-one, cholestanol and other sterol derivatives [4–6]. Historically, tissue injury was attributed primarily to the accumulation of cholestanol in tissues [7]. Current understanding indicates a more complex pathophysiology involving the accumulation of multiple sterol metabolites, disruption of bile acid homeostasis, and altered regulation of cholesterol metabolism in the brain, related to reduced levels of 27-hydroxycholesterol [6, 8].

Because of its pleiotropic manifestations, the disease often requires the involvement of multiple medical specialists during diagnostic process. However, CTX is usually suspected by physicians who have previous experience with the disease or by specialised teams focused on diagnosing metabolic diseases. To facilitate earlier recognition of CTX in clinical practice, a diagnostic “suspicion index” incorporating key systemic and neurological features has been proposed to help identify patients who should undergo targeted biochemical and genetic testing [9].

The first symptoms presenting in patients might be prolonged cholestatic icterus with various degrees of liver impairment shortly after birth. In later childhood or adolescence, the pathognomic signs of the disease are a combination of diarrhoea and cataracts. The diagnostic odyssey usually continues with neurological impairment combined with psychiatric symptoms in adolescent or young adult patients, however CTX is generally considered only after the appearance of tendon xanthomas. Additional clinical signs include osteoporosis and accelerated atherosclerosis [10, 11]. The diagnosis is usually established in patients in their 30 s [12]; however, in milder forms with a late onset [13], the diagnosis could be delayed as late as the 7th decade [13, 14]. The diagnostic approach typically relies on a finding of elevated cholestanol and/or increased urinary excretion of bile alcohols [15, 16]. Due to the application of expensive separation techniques (GC-MS, LC-MS/MS), a diagnosis of CTX is not routinely provided in biochemical laboratories. In addition, several studies have reported CTX patients with atypical biochemical profiles and normal or near-normal serum cholestanol concentrations. Therefore, CTX diagnosis requires integration of clinical findings, biochemical markers, and confirmation by molecular genetic analysis of the *CYP27A1* gene [17, 18].

Therapy for CTX is well established and is based on oral administration of chenodeoxycholic acid, which suppresses bile acid synthesis via negative feedback and reduces accumulation of toxic metabolites [19]. CTX treatment should begin before the onset of neurological manifestations to halt disease progression or reverse some of the manifested symptoms [20].

Due to the rarity and phenotypic variability of CTX, reports of patient cohorts are valuable for improving disease recognition. Most published data originate from Western Europe [12, 21], North America [22], and Asia [23–25], including recent studies from Turkey [18, 26–28], whereas reports from Central and Eastern Europe remain limited [29, 30]. Thanks to our status in metabolic disease diagnostics in our country, we report the first cohort of CTX patients from Slovak Republic. We present data depicting clinical manifestation and diagnostic experience with CTX. In the last year, two new patients were diagnosed using a so far non-conventional approach – a massive parallel sequencing panel. These patients are not reported here due to incomplete data on clinical findings.

## Patients and methods

### Patient selection and diagnostic algorithm

Patients were referred for specialised diagnostics of inborn metabolic diseases based on clinical suspicion of a metabolic disorder. Referral criteria included neurological symptoms, early-onset cataracts, tendon xanthomas, or a combination of these findings. Additional clinical information, including medical history and family genealogy, was collected retrospectively from medical records. Biochemical testing was initiated in patients with a compatible presentation, followed by molecular genetic analysis for confirmation. Because of the limited cohort size, the analysis was descriptive and no formal statistical testing was performed. Patients and their family members provided informed consent for metabolic testing, molecular genetic analysis, and data publication. The study was approved by the Local Ethics Committee (University Hospital Bratislava). This study was conducted in accordance with the ethical principles of the World Medical Association (Declaration of Helsinki).

### Biochemical and genetic analyses

Serum sterols, including cholestanol and 7-dehydrocholesterol, were analyzed by GC-MS using a previously published method [31]. Upper reference values of cholestanol and 7-dehydrocholesterol (16 µmol/l and 0,8 µmol/l, respectively) were taken from the literature [31] and match the values acquired in our cohort of 100 non-CTX individuals (upper reference limit of cholestanol: 14,2 µmol/l, *data not published*). Molecular genetic analysis of the *CYP27A1* gene was performed by Sanger sequencing of all coding exons and flanking intronic regions. Detailed analytical procedures are provided in Supplementary Material 1.

## Results

### Clinical course and laboratory findings in CTX patients

#### Case 1

A 31-year-old woman was referred by a neurologist for laboratory evaluation of inborn errors of metabolism because of suspected cerebrotendinous xanthomatosis, based on an 8-year history of progressive gait disturbance with frequent tripping and instability. Additional neurological symptoms included tremor of her arms, predominantly affecting the right side. Neonatal history was notable for apathy, poor growth, and episodic diarrhoea. Since childhood, she exhibited delayed psychomotor development and speech difficulties and had been evaluated for growth retardation and gastrointestinal symptoms of unknown origin. At 26 years of age, she experienced thrombosis of the right iliac artery and was diagnosed with osteoporosis. Cataracts were not documented in her medical records. Physical examination revealed tendon xanthomas. Psychiatric assessment showed bradypsychism and an infantile behavioural pattern.

EMG of the upper and lower limbs demonstrated mild demyelinating sensorimotor polyneuropathy. Brain MRI performed at 23 years of age revealed Arnold–Chiari malformation type I, leukoencephalopathy of the occipital horns, and an atypically broadened frontal ventricle; similar findings persisted on follow-up imaging two years later. A subsequent MRI at 30 years of age showed mild cerebral atrophy and characteristic lesions in the dentate nuclei and occipital horns. Additional hyperintense lesions were detected in the C2–C4 region and the mid-thoracic segment of the spinal cord.

Based on the combination of neurological symptoms and tendon xanthomas, CTX was suspected and targeted biochemical testing was performed. This revealed markedly elevated serum cholestanol (76,0 µmol/l) and a pronounced increase in 7-dehydrocholesterol (85,4 µmol/l). Molecular genetic analysis of the *CYP27A1* gene subsequently identified two pathogenic variants, c.1016 C > T in exon 5 and c.1183 C > T in exon 6, in the heterozygous state, confirming the diagnosis of CTX.

#### Case 2

The clinical history of the 31-year-old male was relatively short. He was referred for medical genetic counselling because of progressive gait and balance disturbances due to spastic paraparesis, which had been present since the age of 28. Additional symptoms included urinary incontinence and mild facial dysmorphisms, namely an elongated face, coarse lips, and dental diastema. Medical records documented cholestatic icterus of unknown origin during the neonatal period. During adolescence, he had poor school performance, marked hand tremors that later spontaneously resolved, and fast speech during puberty. At 25 years of age, he underwent bilateral cataract surgery. At the time of diagnosis, he was under psychiatric follow-up for social phobia. His medical history also included fractures of the upper and lower limbs.

Brain MRI demonstrated hyperintense signal abnormalities in the periventricular white matter and dentate nuclei, accompanied by diffuse cerebral atrophy. Discrete signal abnormalities with discopathies were noted in the cervical spinal cord. USG revealed fatty infiltrations of the Achilles tendons; however, these were not visually apparent on physical examination. EMG showed a mixed axonal and demyelinating polyneuropathy affecting both upper and lower limbs, with markedly reduced compound muscle action potentials, prolonged distal motor latency, and slowed motor conduction velocities in the tibial and peroneal nerves bilaterally. Dysrhythmic activity was detected on EEG at 27 years of age.

Given the clinical presentation, a metabolic disorder was suspected and a comprehensive metabolic evaluation was performed, including GC–MS analysis of serum sterols. This revealed elevated serum cholestanol (96,0 µmol/l) and markedly increased 7-dehydrocholesterol (77,8 µmol/l). These biochemical findings were consistent with CTX and prompted molecular genetic testing. Sequencing of the *CYP27A1* gene identified a pathogenic frameshift variant, c.819delT in exon 4, in the homozygous state, confirming the diagnosis.

Following confirmation of the molecular diagnosis, a segregation analysis was performed in the family. The patient’s mother and one sister were clinically asymptomatic, exhibited normal serum cholestanol concentrations, and were heterozygous carriers of the identified pathogenic variant. The father was not available for genetic or metabolomic testing.

#### Case 3

The eldest sister of the previously described patient (Case 2) had a medical history notable for psychiatric disturbances, epilepsy, bilateral cataracts, and hepatitis following unspecified drug treatment. At the time of diagnosis, she was 35 years of age. Childhood medical records documented recurrent diarrhoea, attributed to irritable bowel syndrome. At 17 years of age, generalized tonic–clonic seizures and behavioural disturbances were reported, leading to diagnoses of bipolar personality disorder and organic affective disorder. At 24 years of age, she underwent surgical intervention for bilateral cataracts.

At the time of evaluation, neurological findings included episodic cephalgia, cervicopathy, clinically compensated epilepsy, and mild tremors. The Achilles tendons were broadened but without visible typical xanthomas. Her medical history also included two episodes of hepatopathy following cholecystectomy and valproic acid treatment. EEG demonstrated diffusely abnormal activity without specific epileptiform discharges. Brain MRI performed at 35 years of age revealed diffuse cerebral atrophy, mild hypotrophy of the cerebellar vermis, hyperintensities in the dentate nuclei, discrete abnormalities in the globus pallidus and substantia nigra, and mild bilateral supratentorial periventricular leukoencephalopathy.

GC–MS analysis revealed markedly elevated serum cholestanol (86,2 µmol/l) and 7-dehydrocholesterol (73,4 µmol/l) concentrations. Based on the familial context and biochemical findings, targeted molecular genetic testing was performed, confirming the presence of the same pathogenic variant as identified in Case 2 (homozygous pathogenic frameshift variant c.819delT in exon 4).

#### Case 4

A 15-year-old boy was referred for laboratory evaluation because of juvenile cataracts with mild lens clouding and concomitant osteoporosis. Medical records documented a prolonged neonatal cholestatic icterus lasting approximately 3–4 weeks, with complete remission. Upon detailed history-taking, the family reported chronic watery stools present since the first year of life. The patient had no visible tendon xanthomas and no apparent neurological deficits. Physical examination was unremarkable except for mild scoliosis, a slender habitus, and a simple personality profile. The patient’s genealogy was non-contributory.

Metabolic testing was indicated due to cataracts at a young age in conjunction with osteoporosis. The principal findings were significantly elevated cholestanol (69,8 µmol/l) and 7-dehydrocholesterol (29,2 µmol/l) in serum. The CTX diagnosis was confirmed by sequence analysis of the *CYP27A1* gene and the finding of a heterozygous pathogenic variant, c.379 C > T in exon 2 and a pathogenic variant c.1184 + 1G > A in intron 6.

Subsequent family testing demonstrated that the mother was a heterozygous carrier of the c.1184 + 1G > A variant, while the father and the patient’s sister were heterozygous carriers of the c.379 C > T variant. None of the examined relatives showed significant clinical manifestations.

#### Case 5

A 42-year-old woman was diagnosed with CTX following evaluation at our clinic. From the age of 37 years, she experienced progressive balance disturbances, memory impairment, dysphagia, and rapid speech, and also reported migraines. Neonatal icterus was not documented in her medical history. A key diagnostic clue was the presence of bilateral cataracts, which had been surgically treated at 6 years of age. During childhood, she suffered from diarrhoea. In adolescence, a noticeable decline in school performance and mild cognitive deterioration were observed. She was also treated for scoliosis.

On clinical examination, tendon xanthomas were evident. Neurological findings included signs of a central lesion of the left facial nerve, left-sided central hemiparesis, and left-sided limb dystaxia. She was diagnosed with osteopaenia in the lumbar spine and osteoporosis of the hip region.

Brain MRI demonstrated characteristic signal abnormalities in the dentate nuclei, posterior fossa, and cerebellum, accompanied by cerebral and cerebellar atrophy and paraventricular and parietal leukoencephalopathy.

Biochemical analysis revealed markedly elevated serum cholestanol (105,3 µmol/l) and 7-dehydrocholesterol (34,3 µmol/l). Molecular genetic analysis of the *CYP27A1* gene identified a homozygous c.1263 + 5G > A variant in intron 7, which had not been previously published in CTX patients.

Family testing was subsequently performed. Both parents were heterozygous carriers of the identified variant, consistent with autosomal recessive inheritance. Among the tested siblings, two were heterozygous carriers and one was wild-type; all were clinically asymptomatic. Additional asymptomatic heterozygous carriers were identified among aunts and cousins.

#### Case 6

A metabolic disorder was suspected in a 32-year-old male proband by a medical geneticist based on a broad spectrum of symptoms with predominant neurological manifestations. The patient reported “drunken-like” speech, behavioural changes, and memory impairment. At 27 years of age, he noticed progressive thickening of the Achilles tendons. Bilateral cataracts were surgically corrected at the age of 31 years.

Objectively, severe dysarthria, palaeocerebellar syndrome predominantly affecting the right lower limb, marked asynergia, an ataxic–paretic gait, tremor of the right arm, high-arched feet, fixation nystagmus, and hypertrophy of the Achilles tendons were present. Psychiatric findings included obsessive–compulsive disorder, mild intellectual disability, and depression.

EMG demonstrated mild axonal neuropathy. Brain MRI showed pronounced atrophy of the thalamomesencephalic junction, mesencephalon, medulla oblongata, and cerebellar hemispheres. Family history included the death of the patient’s mother due to liver failure, most likely related to alcohol abuse and considered unrelated to CTX.

Biochemical testing revealed markedly elevated serum cholestanol (100,3 µmol/l) and increased 7-dehydrocholesterol (77,4 µmol/l). Molecular genetic analysis of the *CYP27A1* gene identified two pathogenic variants, c.1183 C > T in exon 6 and c.1184 + 1G > A in intron 6, in the heterozygous state.

#### Case 7

A 10-year-old girl was referred to our department by a paediatric metabolic disease specialist. Declining school performance was noted during the clinical evaluation. A neurological assessment suggested possible mild cognitive impairment with attention and memory deficits; however, no typical neurological signs were present. Her difficulties were considered secondary to bilateral cataracts. There was no history of chronic diarrhoea, and prolonged icterus was not reported.

Biochemical testing revealed elevated serum cholestanol (90,6 µmol/l) and 7-dehydrocholesterol (56,9 µmol/l). Molecular genetic analysis identified two previously reported pathogenic variants in the *CYP27A1* gene, c.379 C > T in exon 2 and c.1183 C > T in exon 6, both in the heterozygous state.

Subsequent metabolic and molecular genetic testing of the parents confirmed their carrier status. Of the siblings, three sisters were heterozygous carriers, while the brother carried no pathogenic variant.

#### Case 8

A 5-year-old boy was referred because of psychomotor delay and impaired communication skills. EEG demonstrated epileptiform activity; however, the patient had no clinically apparent seizures. Brain MRI showed no structural abnormalities. In addition to these neurological findings, chronic diarrhoea was reported following targeted history-taking.

Biochemical testing revealed elevated serum cholestanol (46,3 µmol/l) and 7-dehydrocholesterol (23,3 µmol/l). Molecular genetic analysis of the *CYP27A1* gene identified two heterozygous variants, c.379 C > T in exon 2 and c.1263 + 5G > A in intron 7.

Parental testing demonstrated heterozygosity for each of the identified variants, while the patient’s brother was heterozygous for the c.379 C > T variant. Treatment with chenodeoxycholic acid was subsequently initiated, leading to a decrease in serum cholestanol concentrations, improvement in stool consistency, and better communication abilities.

### Summary of clinical manifestations in CTX patients

Patients were diagnosed over an 8-year period. In all cases, the clinical presentation prompted biochemical testing for CTX in the probands and affected individuals. Elevated serum cholestanol and 7-dehydrocholesterol concentrations were detected in all patients.

Premature cataracts represented the most frequent clinical manifestation, with information unavailable in two patients. Neurological and psychiatric features were common among adult patients, with spastic paraparesis and peripheral neuropathy being the predominant neurological findings. In contrast, the youngest patient presented primarily with psychomotor delay and impaired communication. Diarrhoea was reported in several patients, in some cases only after targeted history-taking. Prolonged neonatal cholestatic icterus was documented in a minority of cases; this information was often unavailable in adult patients due to limited knowledge of their perinatal course.

Tendon xanthomas were visually apparent in one patient, while tendon involvement was additionally detected by USG in other cases. This observation may be age-related, as patients with visible xanthomas were older at the time of diagnosis. Hepatic involvement was documented in one patient. Although hepatic manifestations of CTX are typically described in the neonatal period, the finding in our cohort was observed in adulthood; however, alternative contributing factors, including psychiatric medication, could not be excluded.

The spectrum of clinical manifestations observed across the patient cohort is summarized in Table 1. A detailed overview of the identified *CYP27A1* variants and their molecular genetic characteristics is presented in Table 2.

**Table 1:** Clinical findings in patients diagnosed with CTX; y – years at time of diagnosis, m – male, f – female, N/A – not available

Patient	Age of diagnosis/sex	Neurological manifestation	Psychiatric manifestation	Observable xanthomas	Cataract	Diarrhoea	Prolonged neonatal icterus	Hepatopathy
Case 1	31y, f	+	+	+	N/A	N/A	N/A	N/A
Case 2	31y, m	+	+	-	+	N/A	+	-
Case 3	35y, f	+	+	-	+	+	N/A	+
Case 4	15y, m	-	+	-	+	+	+	-
Case 5	42y, f	+	+	+	+	+/-	N/A	-
Case 6	32y, m	+	+	+/-	+	N/A	N/A	N/A
Case 7	10y, f	-	-	-	+	-	-	-
Case 8	5y, m	+	+	-	N/A	+	N/A	N/A

**Table 2:** Gene variants identified in the cases, including nucleotide and protein changes, zygosity, and literature references

Patient	Allele	Nucleotide change (cDNA)	Exon/Intron	dbSNP (rs number)	Zygosity	Protein change	Variant type	Reference
Case 1	1	c.1016 C > T	Exon 5	rs121908102	Heterozygous	p.Thr339Met	Missense pathogenic variant	Reshef et al. 1994 [32]
	2	c.1183 C > T	Exon 6	rs121908096	Heterozygous	p.Arg395Cys	Missense pathogenic variant	Cali et al. 1991 [33]
Case 2	1	c.819delT	Exon 4	rs587778812	Homozygous	p.Asp273Glufs*13	frameshift pathogenic variant	Leitersdorf et al. 1993 [2]
	2	c.819delT	Exon 4	rs587778812	Homozygous	p.Asp273Glufs*13	frameshift pathogenic variant	Leitersdorf et al. 1993 [2]
Case 3	1	c.819delT	Exon 4	rs587778812	Homozygous	p.Asp273Glufs*13	frameshift pathogenic variant	Leitersdorf et al. 1993 [2]
	2	c.819delT	Exon 4	rs587778812	Homozygous	p.Asp273Glufs*13	frameshift pathogenic variant	Leitersdorf et al. 1993 [2]
Case 4	1	c.379 C > T	Exon 2	rs201114717	Heterozygous	p.Arg127Trp	Missense pathogenic variant	Verrips et al. 1999 [34]
	2	c.1184 + 1G > A	Intron 6	rs587778777	Heterozygous	- (Splice donor variant)	Pathogenic splice-site variant	Garuti et al. 1997 [35]
Case 5	1	c.1263 + 5G > A	Intron 7	rs587778784	Homozygous	- (Splice donor variant)	Pathogenic splice-site variant	not published
	2	c.1263 + 5G > A	Intron 7	rs587778784	Homozygous	- (Splice donor variant)	Pathogenic splice-site variant	not published
Case 6	1	c.1183 C > T	Exon 6	rs121908096	Heterozygous	p.Arg395Cys	Missense pathogenic variant	Cali et al. 1991 [33]
	2	c.1184 + 1G > A	Intron 6	rs587778777	Heterozygous	- (Splice donor variant)	Pathogenic splice-site variant	Garuti et al. 1997 [35]
Case 7	1	c.379 C > T	Exon 2	rs201114717	Heterozygous	p.Arg127Trp	Missense pathogenic variant	Verrips et al. 1999 [34]
	2	c.1183 C > T	Exon 6	rs121908096	Heterozygous	p.Arg395Cys	Missense pathogenic variant	Cali et al. 1991 [33]
Case 8	1	c.379 C > T	Exon 2	rs201114717	Heterozygous	p.Arg127Trp	Missense pathogenic variant	Verrips et al. 1999 [34]
	2	c.1263 + 5G > A	Intron 7	rs587778784	Heterozygous	- (Splice donor variant)	Pathogenic splice-site variant	not published

## Discussion

Defects of bile acid synthesis represent a group of very rare inherited metabolic disorders. While several conditions within this group typically present with neonatal cholestasis and hepatopathy, cerebrotendinous xanthomatosis (CTX) is the most widely recognised entity and is increasingly diagnosed. Advances in biochemical and genetic diagnostics have revealed a broader phenotypic spectrum of CTX than previously appreciated, suggesting that the disease may be substantially underdiagnosed.

The estimated frequency of CTX varies considerably depending on the data source. Orphanet reports a prevalence of approximately 1:50 000; however, this estimate appears to be largely based on early observations by Lorincz et al., who identified a pathogenic variant in a single patient and one heterozygous carrier among 115 control individuals [36]. More robust prevalence estimates were provided by Appadurai et al., who analysed ExAC population data (exome sequence data of around 60 000 individuals from the Exome Aggregation Consortium) and estimated the prevalence of CTX in the European population to range between 1:130 000 and 1:460 000 [37]. Subsequent analyses have demonstrated variable geographical distribution, with higher estimated prevalence in Asian populations (1:44 407–1:93 084) and markedly lower prevalence in Finland (1:3 388 767) [38]. Despite these estimates, fewer than 600 patients with CTX have been reported worldwide, strongly suggesting underdiagnosis. Data on the prevalence of CTX in Central and Eastern Europe are currently lacking. Based on the prevalence estimates reported for the European population [37], and considering the population size of Slovakia (approximately 5,4 million), a rough estimate would suggest the presence of at least 40 affected individuals in the country.

In our cohort, we present metabolomic, molecular genetic, and clinical findings that are consistent with previously published data on cerebrotendinous xanthomatosis. Our observations further illustrate the broad and variable phenotypic spectrum of the disease, which extends beyond the classical triad of neurological impairment, cataracts, and tendon xanthomas. Increased awareness of this clinical heterogeneity is essential for earlier recognition of CTX.

In the typical course of CTX, prolonged neonatal or cholestatic icterus is often described as one of the earliest manifestations and may be accompanied by hepatopathy, although progression to liver failure is rare [39, 40].

As observed in our cohort, neonatal hepatic manifestations are frequently overlooked or remain diagnostically unresolved in early medical records, which may delay subsequent consideration of CTX later in life.

Another early clinical feature that may raise suspicion of CTX is chronic diarrhoea, particularly when occurring in combination with cataracts. Diarrhoea typically begins in childhood and may represent the sole clinical manifestation for several years [19, 41]. The underlying mechanism remains incompletely understood. In addition to impaired bile acid synthesis, increased intestinal motility and accelerated intestinal transit, potentially mediated by bile alcohols, have been proposed. This hypothesis is supported by reports demonstrating a correlation between decreased bile alcohol excretion and resolution of diarrhoea [19].

Although urinary bile alcohol analysis was not available in our cohort, diarrhoea was also among the first symptoms to improve after treatment initiation, consistent with published observations.

A cataract is recognised as one of the typical signs of CTX and is reported in over 70% of patients [9]. Cataracts usually occur in the first to the second decade; however, in some patients, they may only be observed in adulthood. Cataracts usually form in both eyes [9, 42]. In our cohort, cataracts were among the most frequent clinical findings, documented in the majority of patients. The age at detection varied considerably, ranging from early childhood to adulthood. Notably, one patient underwent cataract surgery as early as 6 years of age, whereas in other individuals, cataracts were identified or treated in the third decade of life. In several cases, cataracts preceded the onset of neurological symptoms by many years.

Another hallmark of CTX is the presence tendon xanthomas. Their reported prevalence varies across published cohorts. While some studies have described tendon xanthomas in all affected individuals [43], other reports indicate a lower occurrence, approaching 70% [9, 14, 22]. Tendon xanthomas typically develop during the second to third decade of life in patients with CTX [44].

In our cohort, visible tendon xanthomas were observed in 3 out of 8 patients. Three patients were diagnosed at a young age, and tendon xanthomas may not yet have developed at the time of clinical evaluation. In two additional patients without clinically apparent xanthomas, broadened Achilles tendons were noted, and lipid deposits were subsequently identified by USG. These findings suggest that tendon involvement may be missed on routine physical examination when visible xanthomas are absent and subtle changes can be easily overlooked. We therefore propose that, in such cases, ultrasonography should be considered to assess tendon involvement.

Of neurological symptoms, the most prevalent, according to the literature, are motor disturbances and ataxia [45–47], but Parkinsonism, mild/moderate intellectual disability, poor school performance, and epilepsy are also reported [20, 22, 48]. Polyneuropathy is often subclinical and is usually classified as axonal, occasionally demyelinating, or mixed, which represents a common feature of the disease [45].

In our patients, the most frequent neurological manifestations included spastic paraparesis and gait disturbances with dysarthria, which were present in all adult patients. Some patients also exhibited polyneuropathy; however, this was mainly subclinical and diagnosed by EMG.

According to published data, psychiatric manifestations are reported only in a subset of patients with CTX. They usually occur in the context of organic dementia, and several cases of behavioural changes, often manifesting at a younger age, have been described. While uncommon, some patients may predominantly present with psychiatric symptoms, at least in the early course of the disease [14, 20]. There is also a reported case of a young adult with CTX masquerading as attention-deficit/hyperactivity disorder (ADHD) [49]. A purely psychiatric presentation appears to be rare.

During the first two decades of life, psychiatric manifestations such as behavioural changes or intellectual disability should prompt consideration of CTX in the diagnostic work-up and trigger a targeted search for additional disease-specific features. In our cohort, psychiatric symptoms were frequent and were observed in 7 of 8 patients. All adult patients exhibited psychiatric disturbances of varying severity. In one patient (Case 3), psychiatric manifestations represented the dominant clinical feature.

Skeletal involvement represents a recognised, although less frequently discussed, manifestation of CTX. Reduced bone mineral density and osteoporosis, predominantly affecting the vertebrae and long bones, have been reported and may clinically manifest as an increased risk of fractures [10, 50].

Of note, one adolescent patient in our cohort (Case 4) was diagnosed following metabolic assessment prompted by the combination of lens clouding and osteoporosis associated with scoliosis. In total, osteoporosis was documented in three patients in our cohort, including the adolescent case and two adult patients (Cases 1 and 5). Recurrent bone fractures were observed in one additional adult patient (Case 2), although osteoporosis was not formally documented in this individual.

The pathogenesis of skeletal involvement in CTX remains incompletely understood. Proposed mechanisms include impaired vitamin D metabolism, particularly reduced levels of 25-hydroxyvitamin D3 and 24,25-dihydroxyvitamin D3 [10, 51]. Additional contributing factors may include decreased intestinal calcium absorption and increased calcium mobilisation from bone, leading to reduced bone mineral density [52].

Although our data do not allow definitive conclusions regarding the timing of onset of skeletal involvement, the occurrence of osteoporosis in both adolescence and adulthood in our cohort suggests that reduced bone density may represent an underrecognised manifestation of CTX. In clinical practice, the presence of osteoporosis, especially when combined with early-onset cataracts, should raise suspicion of CTX and prompt further diagnostic evaluation.

Several clinical and imaging findings observed in our cohort deviated from the classical presentation of CTX. In addition to the typical triad, some patients presented with early or predominant psychiatric manifestations, while tendon xanthomas were absent or only detectable by imaging in younger individuals. Furthermore, MRI findings showed variability across patients and included cerebral atrophy, white-matter abnormalities, and signal changes in the dentate nuclei described in several cases. These observations highlight that CTX may present with non-classical or incomplete phenotypes, which can contribute to diagnostic delay. Recognising such atypical features may therefore be crucial for earlier diagnosis and timely initiation of treatment.

Bile acid synthesis in humans proceeds through two major pathways, both of which require the activity of sterol 27-hydroxylase. In cerebrotendinous xanthomatosis, deficiency of this enzyme results in impaired synthesis of chenodeoxycholic acid and disruption of normal bile acid homeostasis. As a consequence, sterol intermediates accumulate and are shunted into alternative metabolic pathways, leading to increased production of cholestanol and bile alcohols, which are thought to contribute to tissue injury, particularly within the nervous system [7, 53–55].

Although formal diagnostic guidelines for CTX have not been universally established, current expert recommendations emphasize biochemical testing as a key component of the diagnostic process, with measurement of serum cholestanol representing the most widely used biochemical marker [15]. Similarly, in the Mignarri suspicion index, cholestanol testing is included as a central diagnostic parameter whenever CTX is clinically suspected [9]. However, the sensitivity of cholestanol as a diagnostic marker appears to be lower than previously assumed. Several CTX patients with normal or near-normal serum cholestanol concentrations have been reported [17, 18, 56]. and reduced cholestanol levels have been described in association with glucocorticoid therapy [57] or treatment with ezetimibe [17]. Conversely, elevated cholestanol concentrations have also been reported in conditions other than CTX, such as cholestatic icterus [58] or as a result of certain medications (e.g., propofol) [59], thereby reducing the specificity of cholestanol as a diagnostic biomarker. Additional biomarkers may therefore be useful for improving the sensitivity and specificity of biochemical diagnostics.

In our cohort, serum cholestanol were markedly elevated in all CTX patients, ranging from 46,3 to 105,3 µmol/l. In addition, serum 7-dehydrocholesterol was consistently elevated alongside cholestanol and showed a many-fold increase, approximately 30- to 100-fold (23,3–85,4 µmol/l), compared with reference values (Table 3).

**Table 3:** Biochemical serum parameters in patients with cerebrotendinous xanthomatosis; 7-DHCH – 7-dehydrocholesterol

Patient	Age/sex	Cholestanol [µmol∙l^− 1^]	7-DHC [µmol∙l^− 1^]
Case 1	31y, f	76,0	85,4
Case 2	31y, m	96,0	77,8
Case 3	35y, f	86,2	73,4
Case 4	15y, m	69,8	29,2
Case 5	42y, f	105,3	34,3
Case 6	32y, m	100,3	77,4
Case 7	10y, f	90,6	56,9
Case 8	5y, m	46,3	23,3

**Fig. 1:**
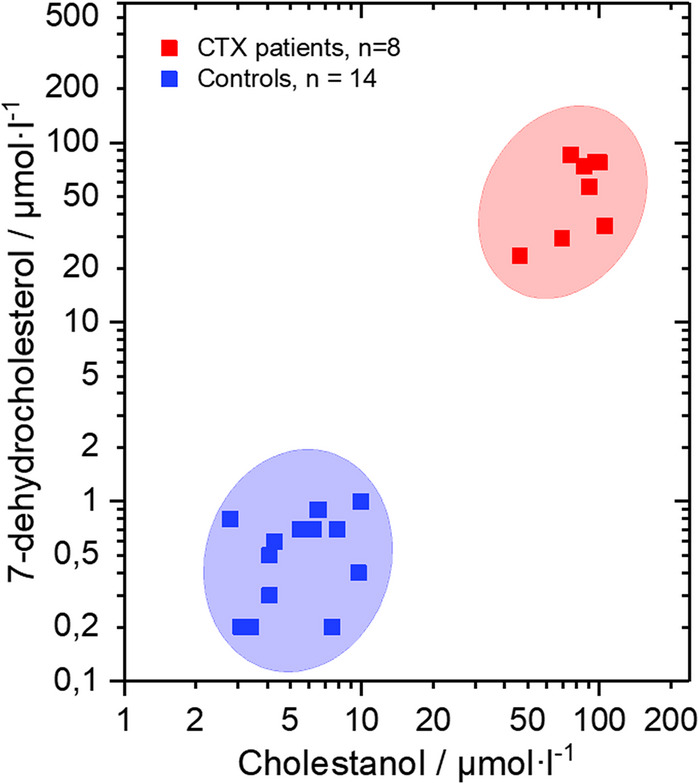
Scatter plot of serum cholestanol and 7-dehydrocholesterol concentrations in CTX patients (red) and healthy controls (blue). CTX patients form a distinct cluster clearly separated from controls, highlighting the diagnostic value of these biochemical markers. Both axes are shown on a logarithmic scale, ellipses indicate the approximate distribution of each group

Those two markers present clear biochemical separation between CTX patients and healthy controls, as illustrated in Fig. 1. The close association between elevated cholestanol and 7-dehydrocholesterol levels, together with their distinct separation from controls, suggests that these parameters reflect disturbances in cholesterol and bile acid synthesis and and may represent useful complementary indicators in the evaluation of the disease.

A practical advantage is that both analytes can be determined within a single GC-MS analytical run. In contrast, some biomarkers require instrumentation that is less widely available, such as LC-MS/MS [60–62], or require analysis of additional biological matrices, such as urine in the case of bile alcohols [63, 64]. On the other hand, because our cohort did not include CTX patients with normal or near-normal serum cholestanol concentrations, it is not possible to confirm whether 7-dehydrocholesterol remains elevated in such individuals. Further studies are needed to assess the usefulness of this two-marker approach, which could represent a more robust biochemical strategy in the biochemical evaluation of CTX.

The relationship between age and cholestanol concentration remains controversial in the literature, with some authors reporting increasing levels from infancy to adulthood in healthy individuals, while others describe relatively stable concentrations across age groups. The highest cholestanol concentration was observed in the oldest patient at the time of diagnosis (42 years, Case 5), whereas the lowest value was measured in the youngest patient (5 years, Case 8). This observation should be interpreted with caution, as no consistent relationship between age and cholestanol concentration was identified, and interindividual variability appeared substantial. Similarly, no clear differences in either cholestanol or 7-dehydrocholesterol concentrations by age or sex were observed in our cohort.

A biochemically based CTX diagnosis must be confirmed by molecular genetic analysis of the *CYP27A1* gene. Sequence analysis of all 9 exons and flanking intronic regions of the *CYP27A1* gene is warranted as the first step in the molecular genetic diagnosis scheme, since most pathogenic variants of CTX have the character of point mutations. In cases where no point mutations are identified despite persistent metabolic suspicion, analysis for deletions or duplications should be considered, as such variants have also been reported in CTX patients.

All the variants established in our patients, except c.1263 + 5G > A, were previously described. Transition c.1263 + 5G > A is not found in any public database of CTX patients; however, the transversion c.1263 + 5G > T has been described as pathogenic variant associated with CTX [35, 65]. We classified the c.1263 + 5G > A variant as pathogenic based on several criteria. Its pathogenicity is supported by in silico prediction tools (VarSome), which classified it as a class 5 variant. The variant was absent from control population databases (gnomAD exomes) and was identified in a patient with typical biochemical findings, including markedly elevated serum cholestanol and 7-dehydrocholesterol levels.

Of note, the variant c.1183 C > T (p.Arg395Cys) is historically among the first *CYP27A1* variants reported in CTX patients [33]. This variant has been identified in multiple populations [36, 38, 66], raising the possibility that it represents a mutational hotspot. In accordance with previous reports, we also observed poor genotype–phenotype correlation among siblings diagnosed with CTX in our cohort.

## Conclusion

Cerebrotendinous xanthomatosis is a rare but treatable disorder caused by a defect in bile acid synthesis. In this study, we present the clinical, biochemical, and molecular genetic findings of a cohort of eight patients diagnosed with CTX in the Slovak Republic.

The clinical presentation was heterogeneous, with neurological manifestations, most commonly spastic paraparesis, frequently accompanied by cataracts and, in some patients, tendon xanthomas or other less typical features. Our findings further illustrate the broad phenotypic spectrum of CTX and underline the diagnostic challenges associated with this condition.

Biochemically, elevated serum cholestanol and 7-dehydrocholesterol consistently distinguished CTX patients from controls and represented robust markers supporting the diagnosis. However, molecular genetic confirmation remains essential for establishing a definitive diagnosis.

The mutational spectrum observed in our cohort largely comprised previously reported *CYP27A1* variants. In addition, we identified the splice-site variant c.1263 + 5G > A, which has not been reported in public CTX databases to date.

Taken together, our data emphasise the importance of integrated clinical, biochemical, and genetic evaluation in the diagnosis of CTX and support increased awareness of this treatable disorder to facilitate earlier recognition and timely initiation of therapy.

## Electronic Supplementary Material

Below is the link to the electronic supplementary material.

Supplementary Material 1

## Data Availability

The data supporting the findings of this study are available from the corresponding author upon reasonable request.

## Declarations

### AI statement

During the preparation of this work, the authors did not use any artificial intelligence tools or services for text generation, data analysis, or figure preparation. The authors take full responsibility for the content of the publication.
