# Supplementary material for: Cerebrotendinous xanthomatosis in Slovak patients – experience with clinical manifestations and diagnostic approaches
Source: Neurogenetics. 2026 Apr 18;27(1):32. doi: 10.1007/s10048-026-00902-6 (PMC13091801; doi:10.1007/s10048-026-00902-6)
Supplement: Supplementary file 1 — Supplementary Material 1 [file 10048_2026_902_MOESM1_ESM.docx]

**Supplementary Material 1**

**Cholestanol and 7-dehydrocholesterol analysis**

The GC/MS sterol analysis included analysis of cholestanol, 7-dehydrocholesterol and other cholesterol related compounds. The analytical method was performed with minor modifications as described in the literature (1), using 5α-cholestane as the internal standard. Briefly, following saponification with ethanolic KOH solution, sterols were extracted with hexane and subsequently derivatized to trimethylsilyl (TMS) ethers using 80 µL of a 1:1 (v/v) mixture of N,O-bis(trimethylsilyl)trifluoroacetamide (BSTFA) and pyridine. Cholestanol and 7-dehydrocholesterol, among other analytes, were separated on a 30 m DB-XLB capillary column (Agilent) under a constant helium flow of 1,0 ml/min, using a TRACE GC Ultra gas chromatograph coupled to an ITQ 1100 ion-trap mass spectrometer (Thermo Scientific) for detection. The total run time was 38 minutes. Quantification was based on analyte-to-internal standard peak area ratios using external calibration curves.

**Molecular genetic analysis of the *CYP27A1* gene**

Genomic DNA was isolated with a QIAamp DNA Blood Mini Kit (Qiagen) from peripheral blood samples taken in standard EDTA test tubes according to the manufacturer’s protocol.

Primers for amplifying nine exons of the *CYP27A1* gene were newly designed using Vector NTI Advance® 11.5 and verified by internet software such as Primer3, Primer-Blast, and SNPCheck. All the encoding exons and their flanking intronic regions were amplified using the primer sets designed by our laboratory. The PCR was tested using a thermal cycler (model SimpliAmp^TM^, Applied Biosystems^TM^). Cycle sequencing was performed on the ABI Prism 3130 genetic analyzer using a BigDye Terminator Cycle Sequencing Reaction Kit (Applied Biosystems). The sequence chromatogram was compared to the published human *CYP27A1* gene sequence (NM_000784.4) using programs Chromas 2.2, Vector NTI Advance® 11.5, and Seqscape. Detected sequence variants were assessed using public databases, including ClinVar, HGMD (Human Gene Mutation Database), and the 1000 Genomes Project, and further evaluated through literature searches in PubMed to determine their potential clinical significance. In silico variant interpretation was additionally performed using the Franklin and VarSome bioinformatic platforms to support pathogenicity classification in accordance with ACMG/AMP guidelines.

**Supplementary references**

1. Waterham H, Duran M. Diagnosis of Inherited Defects of Cholesterol Biosynthesis. In: Blau N, Duran M, Gibson KM, editors. Laboratory Guide to the Methods in Biochemical Genetics [Internet]. Berlin, Heidelberg: Springer; 2008. p. 483–95. Available from: https://doi.org/10.1007/978-3-540-76698-8_24
